# Supplementary material for: A deep learning approach to identify unhealthy advertisements in street view images
Source: Sci Rep. 2021 Mar 1;11:4884. doi: 10.1038/s41598-021-84572-4 (PMC7921635; doi:10.1038/s41598-021-84572-4)
Supplement: Supplementary file 1 — Supplementary Information [file 41598_2021_84572_MOESM1_ESM.pdf]

A deep learning approach to identify unhealthy advertisements in street view images

Gregory Palmer[1,4,+], Mark Green[1,*,+], Emma Boyland[2], Yales Stefano Rios Vasconcelos[3], Rahul Savani[3], and Alex Singleton[1]

[1]Geographic Data Science Lab, Department of Geography and Planning, University of Liverpool, UK
[2]Department of Psychology, University of Liverpool, UK
[3]Department of Computer Science, University of Liverpool, UK
[4]L3S Research Center, Leibniz University Hannover, Germany
[*]mgreen@liverpool.ac.uk
[+]these authors contributed equally to this work

1 Introduction

In this document we provide instructions on how to access the data and machine learning frameworks used for our deep learning approach to identify unhealthy advertisements in street view images. We make our code in the form of python scrips and jupyter notebook files available within our GitHub repository: https://github.com/gjp1203/LIV360SV.

2 Data

2.1 The Liverpool 360 Street View Dataset

In this section we provide instructions for downloading the Liverpool 360 Street View (LIV360SV) dataset. To date the dataset contains 25,349, 360 degree, street-level images collected via cycling with a GoPro Fusion 360 camera (https://gopro.com/en/at/news/the-basics-gopro-fusion).

While there exists an abundance of street-level imagery on platforms such as Google Street View, the recently imposed costs for using Google's API, as well as cases of Google updating terms and conditions to hinder researchers, highlights the need for alternative open sourced solutions. Existing open and crowd sourced street-level images predominately lack the quality of the interactive panoramas found on services such as Google Street View. Images are frequently recorded using dashboard cameras, and as a result have a restricted field of vision. Motivated by these factors we record an open street-level dataset for Liverpool, using a GoPro Fusion 360 camera attached to a member of the team (Mark Green) who cycled along major roads. We follow Mapillary's recommendations (https://help.mapillary.com/hc/en-us/articles/360026122412-GoPro-Fusion-360) for recording street-level images. The camera records front and back images at 0.5 second interval, which we later stitch together using GoPro Fusion Studio. To date our dataset consists of 25,349 street-level images each with GPS location recorded.

We focused on sampling three areas of Liverpool with varying contexts over three different days: (1) City Centre (Jan 14th 2020) - areas characterised by shops and services; (2) North Liverpool (Jan 15th 2020) - areas contain high levels of deprivation; (3) South Liverpool (Jan 18th 2020) - areas include a mixture of affluent populations and diverse ethnic groups. We have uploaded our street level images to Mapillary, which can be viewed here: https://www.mapillary.com/app/org/gdsl_uol?lat=53.39&lng=-2.9&z=11.72&tab=uploads. We used Mapillary because (1) our data could be openly shared alongside other relevant street view images, and (2) Mapillary automatically blurs faces of individuals within images which preserves data privacy concerns and legal requirements.

The images can be downloaded with Mapillary Tools (https://github.com/mapillary/mapillary_tools) using the following command:

```
mapillary_tools download --advanced --by_property key \
--import_path dev/null \
--output_folder './LIV360SV' \
--organization_keys 'I8xRsrajuHHQRf6cdDgDi5' \
--user_name '<Insert Mapillary Username>'
```

To date we have identified 10,106 advertisements within these data, manually classified as food (1335), alcohol (217), gambling (149) and other (8405). A link to download our labelled dataset as a .zip archive with up-to-date md5sum can be found here: https://github.com/gjp1203/LIV360SV#the-liverpool-360-street-view-dataset.

2.2 Advertisement Data

While the Mapillary Vistas includes a billboards category, the dataset does not distinguish different types of advertisements. Further annotations would therefore be necessary to train panoptic scene segmentation networks to differentiate between advertisement types. However, manually annotating segmentation masks is a time consuming task. Instead, we propose to classify advertisements extracted from street level images using a model trained to classify advertisement images.

3 Workflow Components

In this section we describe how setup and utilize each component within our workflow. To recap: we use a seamless scene segmentation network to identify regions within street-level images where advertisements are located. To subsequently classify the extracted advertisements we train a InceptionV3 to differentiate advertisement types using data extracted from Manchester, UK.

3.1 Seamless Scene Segmentation

For extracting advertisements from street level images we use the seamless scene segmentation network introduced by Porzi et al.[1]. The network offers advantages of both semantic segmentation – determining the semantic category that a pixel belongs to – and instance-specific semantic segmentation – the individual object that a pixel belongs to, enabling differentiation between neighbouring entities of the same type. The authors achieve state-of-the-art results on three street-view datasets, including Cityscapes[2], the Indian Driving Dataset[3] and Mapillary Vistas[4]. Instructions for installing Porzi et al.[1]'s seamless scene segmentation implementation network can be found within the following repository: https://github.com/mapillary/seamseg. Upon installing the network images can be segmented using the following command:

```
python3 -m torch.distributed.launch \
--nproc_per_node=1 ./scripts/test_panoptic.py \
--meta ./data/metadata.bin \
./data/config.ini \
./data/seamseg_r50_vistas.tar \
./LIV360SV ./Segmentations \
--raw
```

To provide an idea of the accuracy we compare the mean intersection over union (IoU) for the category billboard against the IoU scores reported on the Mapillary Vistas Validation set by[1]. We depict the IoU as a function on the number of billboard pixels within the ground truth labels, and as expected we find that the IoU increases for larger number of pixels, as larger billboards are easier to identify. The values achieved are in-line with the IoU scores of 0.458 reported across categories on Mapillary Vistas Validation set by[1] (see Table 1).

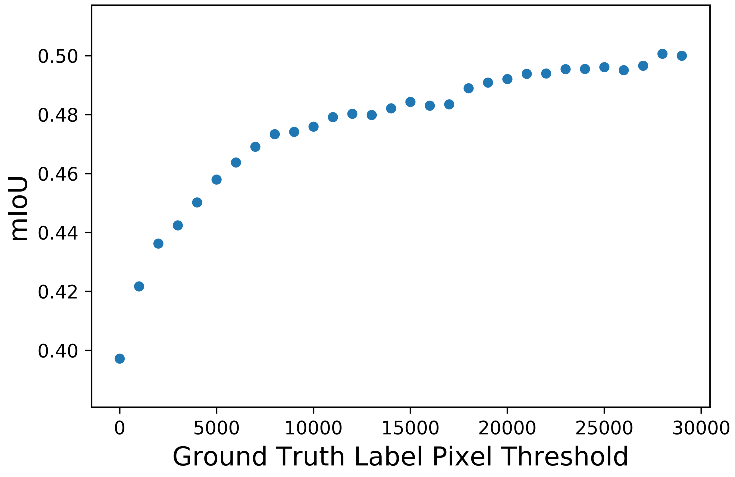

Figure 1. This scatter plot depicts the mean intersection over union (IoU) for the label billboard within validation samples of the mapillary vistas dataset dependent on the number of billboard pixels within the ground truth labels.

Pixel Threshold	mIoU
0	0.3971943030524343
5000	0.4579469165111899
10000	0.47592473477567715
15000	0.4842953521708997
20000	0.49207623930090716
25000	0.4961091670930209
29000	0.4999582723454269

Table 1. mIoU for pixel thresholds.

3.2 Extraction & Preprocessing

Upon identifying the location of an advertisement, we obtain a one hot mask with a filled convex hull using OpenCV's (https://opencv.org/) find and draw contours functionalities. The masks allow us to extract individual advertisements from the original input images. With the remaining content having been masked out during the extraction step we subsequently crop the images. However, given that the final step of our workflow is to pass the extracted items to a classifier trained on advertisement images with a frontal view, we use a Spatial Transformation Network (STN)[5] to transform the extracted items, the majority of which were recorded from a non-frontal view.

The preprocess.py script from our repository can be used to extract and pre-process the images:

```
python3 preprocess.py
```

3.3 Feature Matching Calibration

Upon extracting advertisements we remove spatially proximate near duplicate images using the process described in Section 4.3 in the main paper. We identify suitable feature matching thresholds τ and distance limits d as follows. Our priority is to identify the larger clusters of repeated images within our dataset and calibrate our repeated images detection method accordingly. We argue that finding clusters of sequential repeated images manually is a trivial task compared to manually deleting all repeated images. Therefore we select four large repeated image clusters, three from the extracted food advertisements and one from alcohol:

1. Freddy's Chicken shop (Range 0414_004728 to 0414_004832, 139 images)
2. Hamburger Advertisement (Range 0414_004910 to 0414_004945, 38 images)
3. McDonalds Arch, Edge Lane (Range 7130_001710 to 7130_001825, 81 images)
4. Pig and Whistle Pub (Range 0414_002428 to 0414_002588, 111 images)

We conduct our evaluation using two distance thresholds, $d \in \{10, 100\}$ meters. Therefore, we first obtain the number of matching features for advertisement pairs that are within a distance d of each other, before identifying the sub-graphs for matching features thresholds $\tau \in \{10, 20, 30, ..., 90\}$. In Figure 2b we observe a large number of false positives for lower values for τ. In Figure 2c we also observe that lower thresholds τ result in larger numbers of sub-graphs containing a mix of non-distinct advertisements. However, using large values for τ comes at the expense of the number of true positives. We therefore identify compromise settings, e.g. $\tau \in \{60, 70\}$, and use $\tau = 60$ for our evaluation. In Figure 3 we provide examples of exacted advertisements with a large number of matching features, with Sub-Figures 3a being cases of duplication, while Sub-Figure 3b illustrates a large number of matching features for two distinct advertisements.

3.4 Classification

We classify extracted advertisements using Keras' InceptionV3 (https://keras.io/api/applications/InceptionV3/) implementation. The network is trained using manually labelled extracted samples augmented with the scraped images dataset. To train InceptionV3 open the following jupyter notebook file:

```
jupyter notebook classifier.ipynb
```

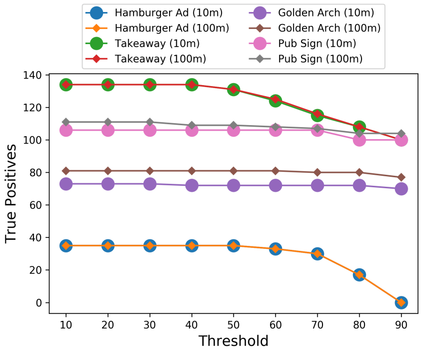

(a) Number of true positives.

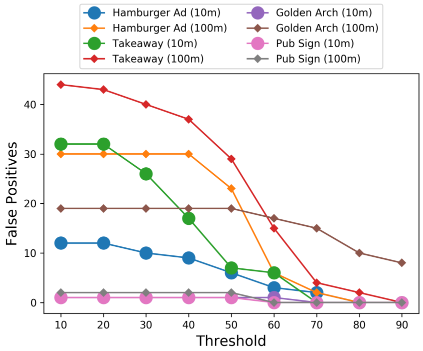

(b) Number of false positives.

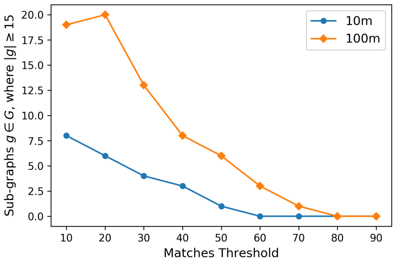

(c) Number of mixed advertisement sub-graphs.

Figure 2

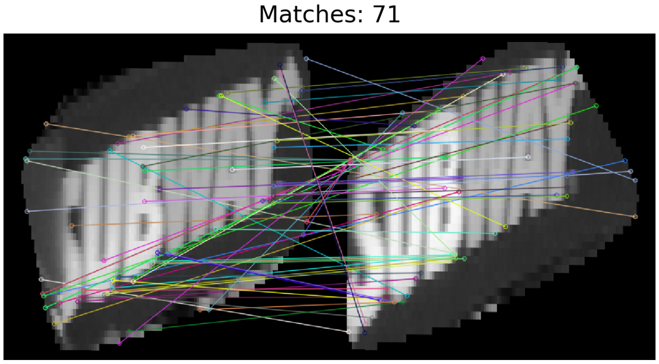

(a) The same advertisement.

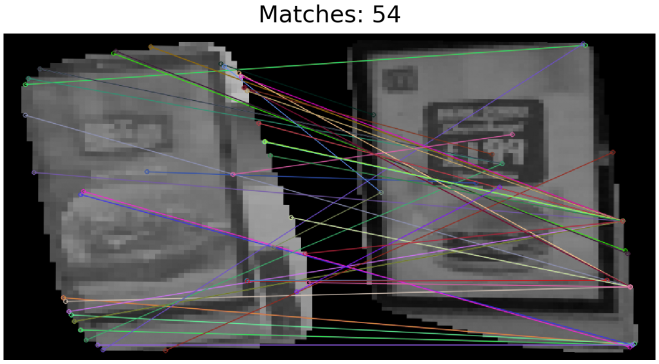

(b) Two distinct advertisements.

Figure 3. Feature matching examples, with Sub-Figure 3a identifying 71 matching features for an advertisement extracted from two street-level-images. However, a large number of matching features are also found for the two distinct advertisements in Sub-Figure 3b.

4 Interpretability

To further interpret the results from Section 5.2, we generate saliency maps to investigate the input features upon which classifications are based. We focus our qualitative evaluation on advertisements extracted from the LIV360SV that belong to brands that are well represented within our training dataset (based advertisements extracted from images recorded in Manchester, UK). Each of these images was correctly predicted to belong to the category food. Through these saliency maps we gain interesting insights. For instance, Sub-Figure 4b shows higher saliency in areas of the image corresponding to the Subway logo in Sub-Figure 4a. Similarly higher saliency can be seen in the top half of Sub-Figure 4d, where the KFC logo is located in Sub-Figure 4c. In contrast for the burger advertisement in Sub-Figure 4e the regions of pixels upon which the prediction is based appear to be more spread-out, as multiple areas are highlighted in Sub-Figure 4f. However, in some instances the regions determining the predictions is less interpretable, as the example for the Costa advertisement in Sub-Figure 4h illustrates. We therefore note that conclusion derived from these saliency maps should be taken with caution. However, we expect more reliable interpretations if the classifier component in Section 5.1 were to be trained using a large-scale data-set, e.g., through building on our current training set by extracting advertisement from additional northern UK cities.

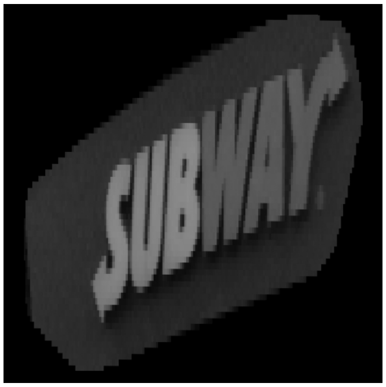

(a) Subway Advertisement

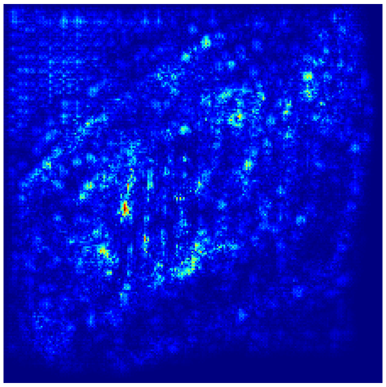

(b) Subway Saliency

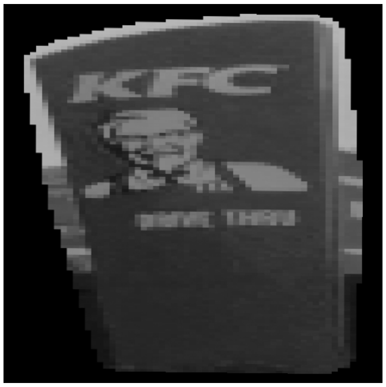

(c) KFC Advertisement

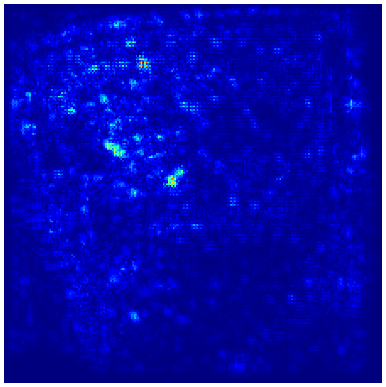

(d) KFC Saliency

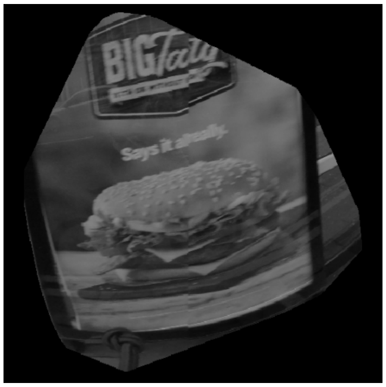

(e) McDonalds Advertisement

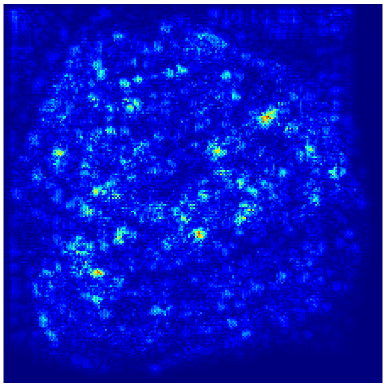

(f) McDonalds Saliency

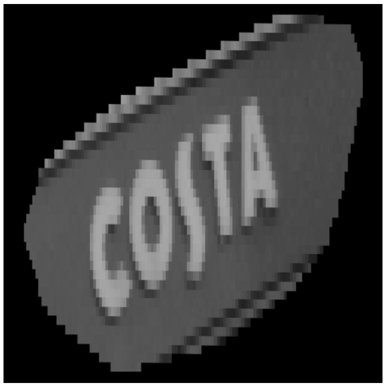

(g) Costa Advertisement

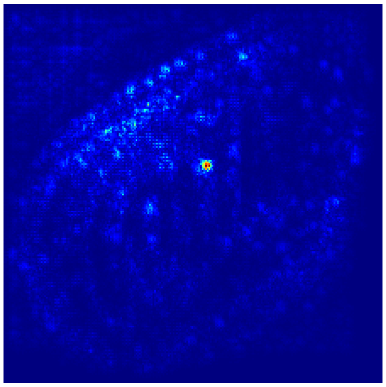

(h) Costa Saliency

Figure 4. Saliency maps examples (right column) for advertisements from well represented fast food brands (left).

5 Using GANs to embed advertisements into street level images

As noted in the discussion section, advertisements extracted from street level imagery are often partially obscured by other real world entities (cars, trees, pedestrians). An approach to improve these issues may be to classify advertisements within street-level imagery augmented with Generative Adversarial Networks (GANs). We propose to embed selected advertisements within street-level imagery through GANs to create additional training data (albeit 'fake data') for model training. To date we can show that advertisements can be successfully integrated into street-level images. We place the advertisement using a STN to transform the image to a target shape. Finally we train GANs to realistically embed the images. We hypothesize that augmenting our collected street view data with these secondary GANs created data will enable the training of an effective model. A link to our latest examples of our placed advertisements as a .zip archive with up-to-date md5sum can be found here: https://github.com/gjp1203/LIV360SV#future-work-using-gans-to-embed-advertisements-into-sv-images.

References

1. Porzi, L., Bulo, S. R., Colovic, A. & Kontschieder, P. Seamless scene segmentation. In *The IEEE Conference on Computer Vision and Pattern Recognition (CVPR)* (2019).

2. Cordts, M. *et al.* The cityscapes dataset for semantic urban scene understanding. In *Proc. of the IEEE Conference on Computer Vision and Pattern Recognition (CVPR)* (2016).

3. Varma, G., Subramanian, A., Namboodiri, A., Chandraker, M. & Jawahar, C. Idd: A dataset for exploring problems of autonomous navigation in unconstrained environments. In *2019 IEEE Winter Conference on Applications of Computer Vision (WACV)*, 1743–1751 (IEEE, 2019).

4. Neuhold, G., Ollmann, T., Rota Bulò, S. & Kontschieder, P. The mapillary vistas dataset for semantic understanding of street scenes. In *International Conference on Computer Vision (ICCV)* (2017).

5. Jaderberg, M., Simonyan, K., Zisserman, A. *et al.* Spatial transformer networks. In *Advances in neural information processing systems*, 2017–2025 (2015).
